# The effects of human immunoglobulin G on enhancing tissue protection and neurobehavioral recovery after traumatic cervical spinal cord injury are mediated through the neurovascular unit

**DOI:** 10.1186/s12974-019-1518-0

**Published:** 2019-07-09

**Authors:** Jonathon Chon Teng Chio, Jian Wang, Anna Badner, James Hong, Vithushan Surendran, Michael G. Fehlings

**Affiliations:** 1 Department of Genetics and Development, Krembil Research Institute, University Health Network, Krembil Discovery Tower, 60 Leonard Avenue, 7KD-430, Toronto, Ontario M5T 2S8 Canada; 2 Institute of Medical Science, University of Toronto, Toronto, Ontario Canada; 3 Sue and Bill Gross Stem Cell Research Centre, University of California, 845 Health Sciences Road, Irvine, CA 92617 USA; 4 Spinal Program, Toronto Western Hospital, University Health Network, Toronto, Ontario Canada; 5 University of Toronto, Toronto, Ontario Canada; 6 Gerry and Tootsie Halbert Chair in Neural Repair and Regeneration, University of Toronto, Toronto, Canada; 7 Krembil Neuroscience Program, Toronto Western Hospital, University Health Network, 399 Bathurst Street, Toronto, Ontario M5T 2S8 Canada

**Keywords:** Spinal cord injury, Human immunoglobulin G, Neuroinflammation, Immunomodulation, Dose-response, Neurovascular unit

## Abstract

**Background:**

Spinal cord injury (SCI) is a condition with few effective treatment options. The blood-spinal cord barrier consists of pericytes, astrocytes, and endothelial cells, which are collectively termed the neurovascular unit. These cells support spinal cord homeostasis by expressing tight junction proteins. Physical trauma to the spinal cord disrupts the barrier, which leads to neuroinflammation by facilitating immune cell migration to the damaged site in a process involving immune cell adhesion. Immunosuppressive strategies, including methylprednisolone (MPSS), have been investigated to treat SCI. However, despite some success, MPSS has the potential to increase a patient’s susceptibility to wound infection and impaired wound healing. Hence, immunomodulation may be a more attractive approach than immunosuppression. Approved for modulating neuroinflammation in certain disorders, including Guillain-Barre syndrome, intravenous administration of human immunoglobulin G (hIgG) has shown promise in the setting of experimental SCI, though the optimal dose and mechanism of action remain undetermined.

**Methods:**

Female adult Wistar rats were subjected to moderate-severe clip compression injury (35 g) at the C7-T1 level and randomized to receive a single intravenous (IV) bolus of hIgG (0.02, 0.2, 0.4, 1, 2 g/kg), MPSS (0.03 g/kg), or control buffer at 15 min post-SCI. At 24 h and 6 weeks post-SCI, molecular, histological, and neurobehavioral effects of hIgG were analyzed.

**Results:**

At 24 h post-injury, human immunoglobulin G co-localized with spinal cord pericytes, astrocytes, and vessels. hIgG (2 g/kg) protected the spinal cord neurovasculature after SCI by increasing tight junction protein expression and reducing inflammatory enzyme expression. Improvements in vascular integrity were associated with changes in spinal cord inflammation. Interestingly, hIgG (2 g/kg) increased serum expression of inflammatory cytokines and co-localized (without decreasing protein expression) with spinal cord vascular cell adhesion molecule-1, a protein used by immune cells to enter into inflamed tissue. Acute molecular benefits of hIgG (2 g/kg) led to greater tissue preservation, functional blood flow, and neurobehavioral recovery at 6 weeks post-SCI. Importantly, the effects of hIgG (2 g/kg) were superior to control buffer and hIgG (0.4 g/kg), and comparable with MPSS (0.03 g/kg).

**Conclusions:**

hIgG (2 g/kg) is a promising therapeutic approach to mitigate secondary pathology in SCI through antagonizing immune cell infiltration at the level of the neurovascular unit.

## Background

Despite significant medical advances, spinal cord injury (SCI) continues to be a debilitating neurological condition. SCI pathophysiology is divided into two stages [1], beginning with damage from the initial physical injury causing an immediate structural disturbance. This initial physical insult is exacerbated during the second stage by infiltration of immune cells into the injured spinal cord (hereby referred to as neuroinflammation), which is associated with damage to the blood-spinal cord barrier (BSCB) and loss of tight junction (TJ) proteins. To target neuroinflammation, systemic immunosuppressive strategies, including methylprednisolone (MPSS), have been successfully used following the initial physical insult to improve functional outcomes in pre-clinical and clinical SCI studies [2–6]. However, recent research suggests that these therapies may be suboptimal, as patients develop systemic immune deficiency [7, 8] and the beneficial aspects of neuroinflammation, necessary for regeneration and remyelination, become limited [9]. Therefore, immunomodulation is deemed a more attractive approach than systemic immunosuppression to target the neuroinflammatory response.

Human immunoglobulin G (hIgG) is approved by the Food and Drug Administration to treat both immunodeficiency and autoimmune conditions [10, 11]. Despite the wide use of hIgG in the clinic, the mechanism of action remains uncertain [10]. Numerous studies have reported a diverse array of effects, including B and T cell trafficking, neutralization of autoantibodies, modulation of the complement cascade, and cytokine production. These effects are mediated by both F_c_ and F_(ab)2_ components of hIgG. Although previous research has demonstrated hIgG-induced increases in functional recovery by reducing neuroinflammation in pre-clinical models of SCI, the method of administration [12] and models used have not been clinically relevant [13, 14]. Further, it is unknown whether the beneficial effects of hIgG following SCI are dose-dependent. Given these gaps in knowledge, we used a well-characterized clip compression-contusion SCI model with a minimally invasive administration method to identify the optimal dose of hIgG administration and examined the immunomodulatory effects on the immune response after SCI. We hypothesized that early treatment with hIgG would immunomodulate spinal cord inflammatory cell populations after traumatic cervical SCI, in part by enhancing the integrity of the BSCB. This work builds on our prior publication [15] by demonstrating that high-dose hIgG (2 g/kg) is significantly more effective than our previously reported hIgG dose (0.4 g/kg) at modulating the acute neuroinflammatory response. Further, the present study shows that hIgG has protective effects that are mediated through the spinal cord neurovascular unit.

## Materials and methods

### Experimental and control groups

A total of 124 female adult Wistar rats (250–300 g) from Charles River were used in this study. Of these rats, 52 were used for dose-response studies to investigate the molecular and biochemical changes, 24 to examine the changes in tissue morphology and histology, and 48 to assess the neurobehavioral recovery and tissue preservation. All procedures followed the animal use protocol approved by the Animal Use Committee at the University Health Network. Rats were randomly assigned to sham (receiving laminectomy only) or SCI groups. For rats in the SCI group, a well-characterized model of injury (35-g modified aneurysm clip applied extradurally at the C7-T1 level for 60 seconds (s) to cause a moderate-severe SCI) was used [16, 17]. Animals were given 1 ml of buprenorphine (0.03 mg/kg) and 5 ml of saline immediately following surgery. Subsequently, buprenorphine and saline were given twice a day up to 3 and 7 days, respectively.

**Fig. 1:**
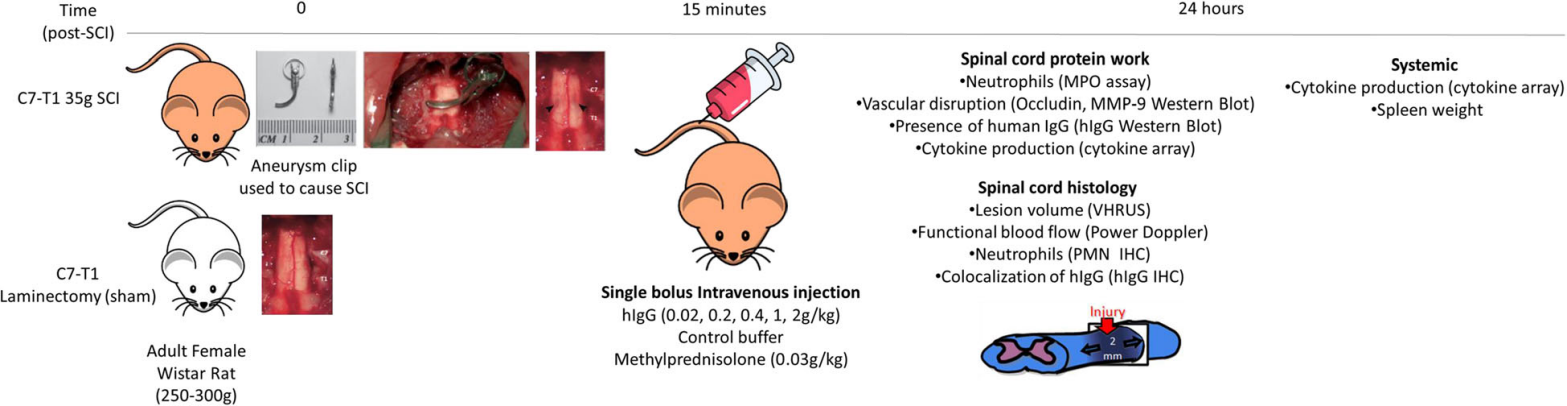
Illustration depicting the experiments and protocol applied. Adult female Wistar rats (250 to 300 g) received either a C7-T1 35g clip-compression injury or sham surgery (C7-T1 laminectomy). At 15 mins post-injury, a single bolus of hIgG (0.02, 0.2, 0.4, 1, or 2 g/kg), control buffer, and MPSS (immunosuppressant; 0.03 g/kg) were administered to the injured rats through the tail vein. At 24 hours (h) post-SCI, the spinal cord was collected from the rats for various assays to analyze the local acute inflammatory response. Another group of rats was used for VHRUS, Power Doppler, and immunohistochemistry (IHC) analyses. For protein and histology work, 2 cm of the spinal cord centered at the epicenter was obtained

hIgG (100 mg/ml) and control buffer were provided by Shire/Baxalta (USA). At 15 min post-SCI, a single bolus of hIgG was intravenously administered at various doses (0.02 g/kg (*n* = 9), 0.2 g/kg (*n* = 6), 0.4 g/kg (*n* = 12; *n* = 6 for biochemical analysis, 6 for ultrasound and histology), 1 g/kg (*n* = 6), 2 g/kg (*n* = 12; *n* = 6 for biochemical analysis, 6 for ultrasound and histology)). Control buffer (*n* = 12; *n* = 6 for biochemical analysis, 6 for ultrasound and histology) or methylprednisolone (MPSS, 0.03 g/kg) (*n* = 7) were injected in an identical fashion as negative and positive controls, respectively. The dosage of MPSS given was based on the reported dosage for human SCI patients [18], whereas dosages of hIgG corresponded to those currently used in the clinic [10]. For all studies, randomization was achieved through prepared lists, with treatment allocation remaining concealed from the investigators conducting the surgery or experiments. There was no noticeable difference in the general behavior, appearance, or mortality rate between the animals treated with hIgG, control buffer, or MPSS. Experimental schematic of acute studies is shown in Fig. 1.

### Biochemical analyses

Biochemical analyses were performed on 52 rats following SCI (hIgG 0.02 g/kg (*n* = 9), hIgG 0.2 g/kg (*n* = 6), hIgG 0.4 g/kg (*n* = 6), hIgG 1 g/kg (*n* = 6), hIgG 2 g/kg (*n* = 6), control buffer (*n* = 6), MPSS (0.03 g/kg) (*n* = 7), and sham (*n* = 6)). Rats were sacrificed at 24 h for various assays (western blot, proteome profiler, and myeloperoxidase (MPO) assay). Each animal was deeply anesthetized with isofluorane and transcardially perfused with 180 ml of ice-cold 1× phosphate-buffered saline (PBS). The spleens were subsequently removed and weighed. A 2-cm length of the spinal cord, centered at the injury epicenter, was dissected. The meninges were removed, and the cord was immediately frozen with dry ice. For tissue processing, the spinal cords were thawed and homogenized with 1× PBS without calcium and magnesium (Wisent) containing 1:1000 protease and phosphatase inhibitors (100×) (Thermo Scientific). The homogenate was split for the respective assays and frozen in liquid nitrogen.

#### Myeloperoxidase activity assay

MPO activity was determined using a MPO fluorometric kit available from Assay Designs (Enzo Life Sciences), according to the manufacturer’s instructions. To prepare the homogenate, the cellular membranes were disrupted and the blood was removed by homogenizing the spinal cord tissue in the provided homogenization buffer (without detergent) containing 10 mM *N*-ethylmaleimide. The samples were then centrifuged at 4 °C at 12,000*g* for 20 mins, and the supernatant was removed. MPO was released from the granules in pelleted material by sonicating in solubilization buffer containing 0.5% of the detergent hexadecyltrimethylammonium (*w*/*v*) and also by exposing the mixture to two freeze/thaw cycles. The samples were then centrifuged at 8000*g* for 20 mins at 4 °C. A Perkin-Elmer plate reader measured the fluorescence intensity of the resultant supernatants, with excitation wavelength at 530 nm and emission wavelength at 590 nm. A calibration curve run concurrently with the samples was used to determine the MPO activity from the measured relative fluorescence intensity.

#### Western blot

Homogenate was solubilized in 200 μl of RIPA buffer (25 mM Tris-HCl pH 7.6, 150 mM NaCl, 1% NP-40, 1% sodium deoxycholate, 0.1% sodium dodecyl sulfate; Thermo Fisher Scientific) containing a 100× cocktail of phosphatase and protease inhibitors and EDTA (Thermo Scientific). Samples were spun down at 14,140*g* at 4 °C for 20 mins. The protein concentration of each sample was measured using the bicinchoninic acid assay (Thermo Scientific). To run the western blot, all samples had 20 μg of total protein and were separated using sodium dodecyl sulfate polyacrylamide gel electrophoresis with 4% stacking-12% resolving gel. Following electrophoresis, the gel was transferred onto a nitrocellulose membrane for 1 h at 100 V. The membrane was then washed in 0.2% Tween Tris-buffered saline (TBS-T) and blocked for 1 h in TBS-T + 5% milk (*w*/*v*). The primary antibodies used (concentration, incubation time, purpose) are listed in Table 1. The secondary antibody incubation step was accomplished at room temperature for 1 h with 1:3000 goat anti-rabbit HRP (Biorad). The membrane was washed with TBS-T three times (10 mins each time) between each antibody incubation step. Enhanced chemiluminescence (ECL; Perkin Elmer) substrate for horseradish peroxidase (HRP) was used to detect the signal. Densitometry analysis was accomplished using ImageJ software.

**Table 1:** List of primary antibodies used for western blot, concentration, incubation parameters, and purpose

Antibody	Concentration	Incubation time and conditions	Application
Rabbit anti-rat Iba-1; Wako Laboratory; Chemicals 019-19741	1:1000	Overnight; 4 °C	Resident microglia activation
Rabbit anti-rat MMP-9; Millipore; AB19016	1:3000	Overnight; 4 °C	Blood-spinal cord barrier degradation
Rabbit anti-hIgG; Abcam; AB109849	1:5000	Overnight; 4 °C	Human IgG in the spinal cord
Goat anti-rat occludin; sc-8144	1:500	Overnight; 4 °C	Blood spinal-cord barrier integrity
Mouse anti-rat ZO-1; MABT11	1:250	Overnight; 4 °C	Blood spinal-cord barrier integrity
Rabbit anti-rat VCAM-1; Abcam; AB134047	1:4000	Overnight; 4 °C	Vascular adhesion molecule
Mouse anti-rat β-actin HRP conjugated; ab49900	1:10000	1.5 h; room temperature	Loading control

#### Proteome profiler

Nine rats were used for the proteome profiler (control buffer *n* = 3, sham *n* = 3, hIgG 2 g/kg *n* = 3). The expression of rat cytokines in the spinal cord (after different treatment administrations) was detected using a commercially available proteome profiler rat cytokine array kit (Panel A) (R&D systems) according to the manufacturer’s instructions and with 100 μg protein per sample.

### Histological, functional blood flow, and immunohistochemical analysis

For histology and functional blood flow analyses, 24 rats (control buffer *n* = 6, hIgG 0.4 g/kg *n* = 6, hIgG 2 g/kg *n* = 6, sham *n* = 6) were analyzed with in vivo very high-resolution ultrasound (VHRUS) and Power Doppler imaging, as previously described [19]. At 24 h after injury, the animals were anesthetized using isofluorane and placed on an imaging platform (Vevo Imaging Station, VisualSonics, Toronto, Ontario, Canada, http://www.visualsonics.com) with a custom-made stabilization frame. The injury was exposed with a midline incision and retraction of the paraspinal muscle layers. Ultrasound gel (scanning gel, Medi-Inn, www.medi-inn.com) was placed on the dura mater and scanned with the VHRUS probe (44 MHz, Vevo 770, VisualSonics) in three-dimensional (3D) B-mode. The 3D B-mode scans were analyzed using ImageJ software as previously described [19], with minor modifications. Briefly, the bright pixels were delineated by one independent blinded observer within 19 central sagittal image slices, and these slices were used to generate a reproducible lesion volume with the TrakEM2 plugin. For Power Doppler imaging, functional blood flow was quantified by measuring the percent area of power Doppler signal (color threshold positive pixels) in a standardized spinal cord region within the image slices. The scan speed was 2.0 mm/s, and the wall filter was 2.5 mm/s.

Subsequently, the animals were deeply anesthetized with isofluorane and perfused with 60 ml of ice-cold 1× PBS and 180 ml of paraformaldehyde (Sigma Aldrich) (4% *w*/*v* in 1× PBS, pH 7.4). Two centimeters of the spinal cord (centered at the injury epicenter) was dissected and post-fixed for 5 h with 10% sucrose (Bioshop) and 4% PFA-PBS solution. The spinal cord was subsequently cryoprotected in 30% sucrose PBS solution. The spinal cord tissue (2 cm centered at the injury epicenter) was embedded in M1 media (Thermo Fisher Scientific) and stored at − 80 °C. The spinal cords were cryosectioned at 20 μm thickness.

The frozen tissue sections were stained with various antibodies diluted with blocking solution made of 1× PBS containing 5% *w*/*v* milk (Bioshop), 1% *w*/*v* bovine serum albumin (Sigma Aldrich), and 0.03% Triton X-100 (Thermo Scientific). Antibody, concentration, and purpose are listed in Table 2. The tissue sections were blocked for 1 h in room temperature with a blocking solution. Secondary antibody alone (no primary antibody) served as the negative control. Washing steps after antibody application were accomplished with 1× PBS washes three times each for 10 min, with the last wash also containing 1:1000 DAPI (4′,6-diamidino-2-phenylindole) to counterstain for the nuclei. The slides were mounted onto the coverslips using Mowiol (Sigma Aldrich).

**Table 2:** List of primary antibodies used for immunohistochemistry, concentration, incubation parameters, and purpose

Antibody	Concentration	Incubation time and conditions	Application
Rabbit anti-rat polymorphonuclear cells (PMN) conjugated with FITC (Cedarlane; CLFAD51140)	1:100	Overnight; 4 °C	Neutrophil infiltration
Rabbit anti-rat Iba-1 (Wako Laboratory Chemicals; 019-19741)	1:300	Overnight; 4 °C	Resident microglia
Lycopersicon esculentum (tomato) (FITC conjugated; Sigma; L0401)	1:500	Overnight; 4 °C	Blood vessel
Rabbit anti-GFAP (Millipore; AB5804)	1:500	Overnight; 4 °C	Astrocytes
Mouse anti-NG2 chondroitin sulfate proteoglycan antibody (Millipore; AB5384)	1:250	Overnight; 4 °C	Pericytes
Rabbit PDGF receptor beta antibody [Y92] (Abcam; ab32570)	1:250	Overnight; 4 °C	Pericytes
Rabbit anti-rat VCAM-1; Abcam; AB134047	1:250	Overnight; 4 °C	Vascular adhesion molecule
Goat anti-human IgG (H+L) (cross-adsorbed secondary antibody AlexFluor 568) (Fisher-Invitrogen)	1:150	2 h; room temperature	Human IgG

#### Stereological quantification of neutrophil infiltration and cellular localization/tissue distribution of IgG at 24 h post-SCI

For stereological quantification, tissue was systematically sampled at every 240 μm over a distance of 4800 μm, which was centered at the lesion epicenter of each animal. Neutrophils were quantified stereologically using the Zeiss Axioplan Deconvolution Microscope (with AxioVision), with neutrophils being PMN^+^/DAPI^+^. For co-localization of hIgG with rat spinal cord blood vessels, microglia, neutrophils, astrocytes, pericytes, and VCAM-1, tissue was imaged at the lesion epicenter using confocal microscopy (Nikon Eclipse Ti) at × 120.

### Tissue preservation analysis

At 6 weeks post-SCI, the spinal cords were perfused in an identical fashion as described for histological, functional blood flow, and immunohistochemical analyses. The tissue sections from 22 animals (control buffer *n* = 6, hIgG (0.4 g/kg) *n* = 6, hIgG (2 g/kg) *n* = 6, sham *n* = 4) were systematically sampled at every 240 μm over a distance of 3600 μm (1800 μm rostral and caudal of the epicenter). The tissue sections were stained with luxol fast blue (LFB) overnight at 56 °C. Hematoxylin and eosin (H&E) was used to stain for the cell nuclei and counterstain, respectively. Unbiased measurements were made with a Cavalieri probe (Stereoinvestigator, MBF Bioscience, Williston, VT) for total tissue, gray matter, white matter, lesion, and cavity volumes.

### Neurobehavioral assessment of functional recovery

All neurobehavioral assessments were performed and analyzed by examiners blinded to the treatment groups. A total of 42 animals (control buffer *n* = 12, hIgG (0.4 g/kg) *n* = 12, hIgG (2 g/kg) *n* = 12, sham *n* = 6) were used for neurobehavioral assessments, which were performed once per week for 6 weeks post-SCI. This neurobehavioral assessment paradigm has been previously used in our laboratory [15, 20]. Hindlimb function was tested using the 21-point open-field Basso-Beattie-Bresnahan (BBB) Locomotor Scale. To assess the whole-body and trunk motor function, the inclined plane test was performed. The animals were placed on a flat plane surface, and each subsequent trial increased the angle of the plane at increments of 5°. The rats were placed with their body axis parallel to the plane. The rats were required to maintain their body position on the plane for 5 s for 3 of 5 attempts. Forelimb strength was tested using a grip strength meter. For this strength test, the animals were held by the hindlimbs and lower abdomen and drawn backwards at a consistent speed within reach of a metal rung connected to the strength apparatus. The animals grasp the rung reflexively, and a strength meter measures the maximal force achieved when the grip was broken. Strength was averaged over 5 successful grasps. To test for neuropathic pain, the animals were subjected to the tail flick test. Using a specialized device, the rats were wrapped in a blanket while the tail was left exposed to a beam of light, which served as a heat source. The time elapsed before the rat flicked its tail in response to heat was recorded.

### Statistical analysis

Data are reported as mean ± standard error of the mean (SEM). For biochemical, immunohistochemical, molecular, and ultrasound analyses, comparisons were based on a one-way ANOVA (analysis of variance) and Tukey’s post-hoc test, with multiple *t* tests (Holm-Sidak method) performed for proteome profiler. Pearson’s correlation coefficient was used to determine ZO-1, occludin, and MMP-9 relations. For neurobehavioral recovery and tissue preservation, two-way ANOVA and Tukey’s post-hoc tests were performed. All statistical analyses were performed using Prism 6.0 Software (GraphPad Version 6.01). Differences were considered significant if *p* < 0.05.

## Results

### Cellular localization and tissue distribution of hIgG in the spinal cord 24 h post-SCI

**Fig. 2:**
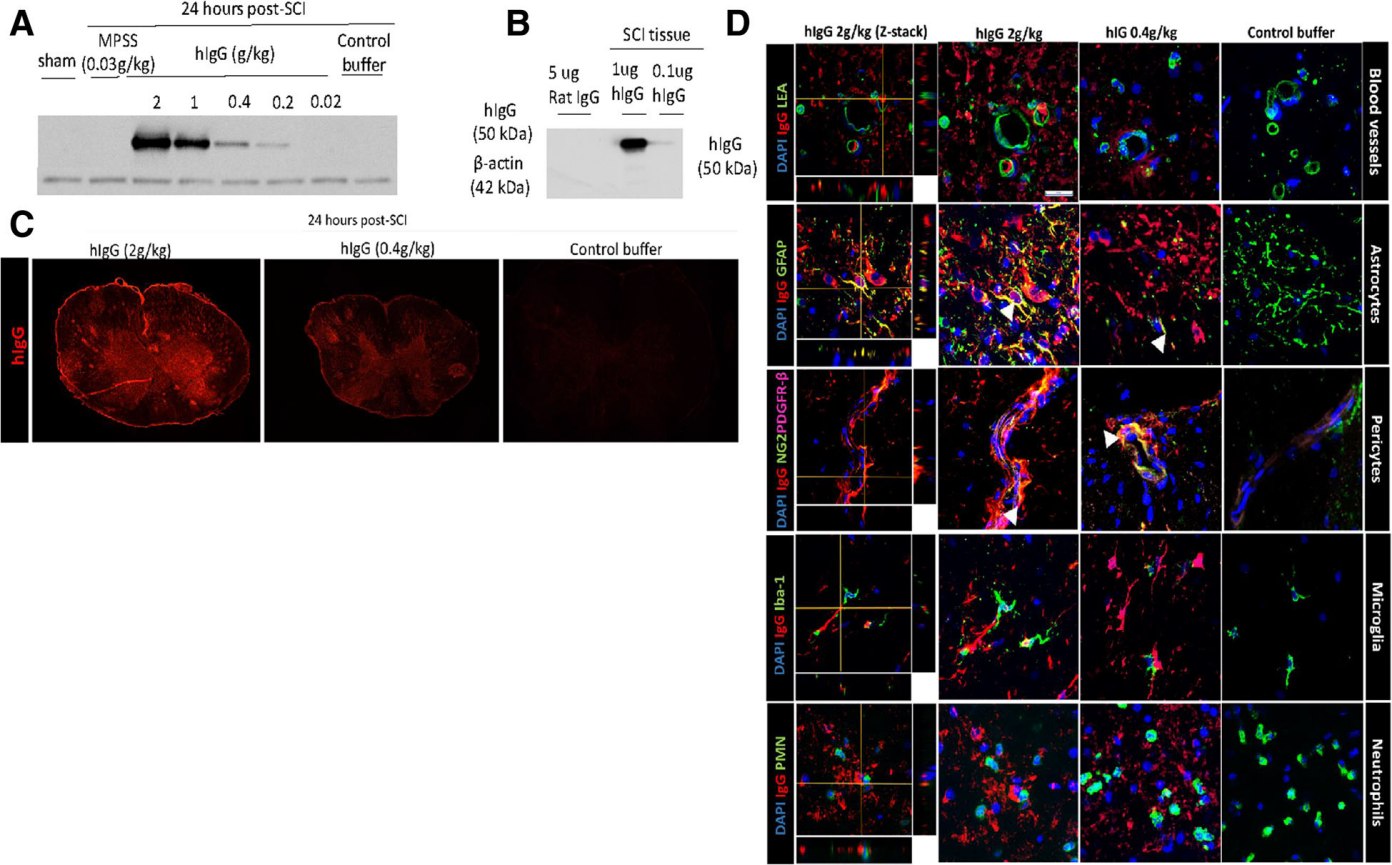
hIgG crosses the BSCB at 24 h post-SCI. The presence of hIgG in the spinal cord was determined in order to evaluate if it is associated with the immunomodulatory effects of hIgG after SCI. **a** Western blot was performed to determine the levels of hIgG in the spinal cord after different treatments. **b** Relative to control buffer and hIgG (0.4 g/kg), high-dose hIgG (1 and 2 g/kg) administered at 15 min post-SCI increased the hIgG levels in the injured spinal cord at 24 h post-SCI. **c** Western blot results are complemented with immunohistochemistry, as in the representative fluorescence images there is a stronger staining of hIgG in the spinal cords treated with hIgG (2 g/kg) as compared to hIgG (0.4 g/kg) and control buffer. **d** The blood vessels (LEA), neutrophils (PMN), microglia (Iba-1), and astrocytes (GFAP) are stained green, pericytes (NG2 green, PDGFR-β: fuchsia), and hIgG are stained red. Confocal images (× 120) demonstrate that hIgG crosses the compromised BSCB in injured animals. Representative images demonstrate hIgG in the vicinity of resident microglia and neutrophils, but hIgG colocalizes with the hIgG, astrocytes, pericytes, and blood vessels. For both **c** and **d**, a hIgG-positive signal was not observed in the spinal cord of rats injected with control buffer. **d** Scale bars represent 10 μm in length

To determine if the presence of hIgG in the spinal cord is necessary for dose-dependent immunomodulatory effects after SCI, a western blot for hIgG was completed. Previous work has shown [13, 15] that intravenously administered hIgG enters the injured cord through the compromised BSCB. Yet, it is unclear if the penetration of hIgG into the injured spinal cord is a prerequisite for hIgG’s observed effects. As demonstrated in the hIgG western blot, there was a dosage-dependent increase in hIgG that reached the injured spinal cord at 24 h post-SCI (Fig. 2a), while there was no cross-reactivity with rat immunoglobulin G (Fig. 2b).

To complement the hIgG western blot, IHC was performed to detect hIgG levels at 24 h post-SCI as well as potential co-localization with other cell types in the rat spinal cord. Epifluorescent images were acquired using a × 20 objective at the lesion epicenter. At 24 h after injection, the presence of hIgG increased in a dose-dependent manner in rats treated with hIgG and was absent in rats treated with the control buffer (Fig. 2c). hIgG was found inside and outside the spinal cord vasculature (marked by FITC LEA positive staining) in the spinal cord of injured animals; re-affirming that a compromised BSCB after SCI enables hIgG to enter into the injured spinal cord (Fig. 2d). Further, as demonstrated with representative confocal images (× 120), hIgG surrounded the resident microglia, neutrophils, and blood vessels, as indicated by FITC-positive PMN, Iba-1, and LEA staining. Importantly, hIgG co-localized with rat astrocytes, pericytes, and adhesion molecule, VCAM-1. Although our previous publication showed co-localization with rat astrocytes [15], we extended these findings to demonstrate potential interactions between hIgG and the spinal cord neurovascular unit (astrocytes and pericytes) as well as immune cell adhesion molecules [21]. Further, while Iba-1 stains for monocytes and microglia, Iba-1-positive cells were likely microglia, as monocytes infiltrate the injured spinal cord at 3 days post-SCI [22].

### hIgG decreases damage to the spinal cord vasculature after SCI

Interactions between hIgG and the spinal cord neurovascular unit prompted us to evaluate the potential effects on the BSCB. In SCI, physical trauma decreases the expression of tight junction proteins, which are needed to maintain BSCB integrity [23]. In addition, the integrity of the BSCB can be further compromised by upregulation of inflammatory enzymes that degrade the components [24]. Abnormalities in the permeability and function of the BSCB after SCI are directly linked with infiltration of immune cells, which exacerbate damage from the primary injury. A key inflammatory enzyme is matrix metalloproteinase-9 (MMP-9), which is a zinc- and calcium-dependent endopeptidase produced by neutrophils and microglia after SCI to degrade the major components of the basal lamina and tight junctions. MMP-9 facilitates the entrance of immune cells and initiates SCI-induced secondary damage [24, 25]. We have previously shown that hIgG (0.4 g/kg) reduces MMP-9 expression at 24 h post-SCI [15]. Western blots were performed to evaluate dose-dependent effects of hIgG on the expression of MMP-9 at 24 h post-SCI and consequential effects on BSCB integrity (spinal cord protein expression of ZO-1 and occludin).

**Fig. 3:**
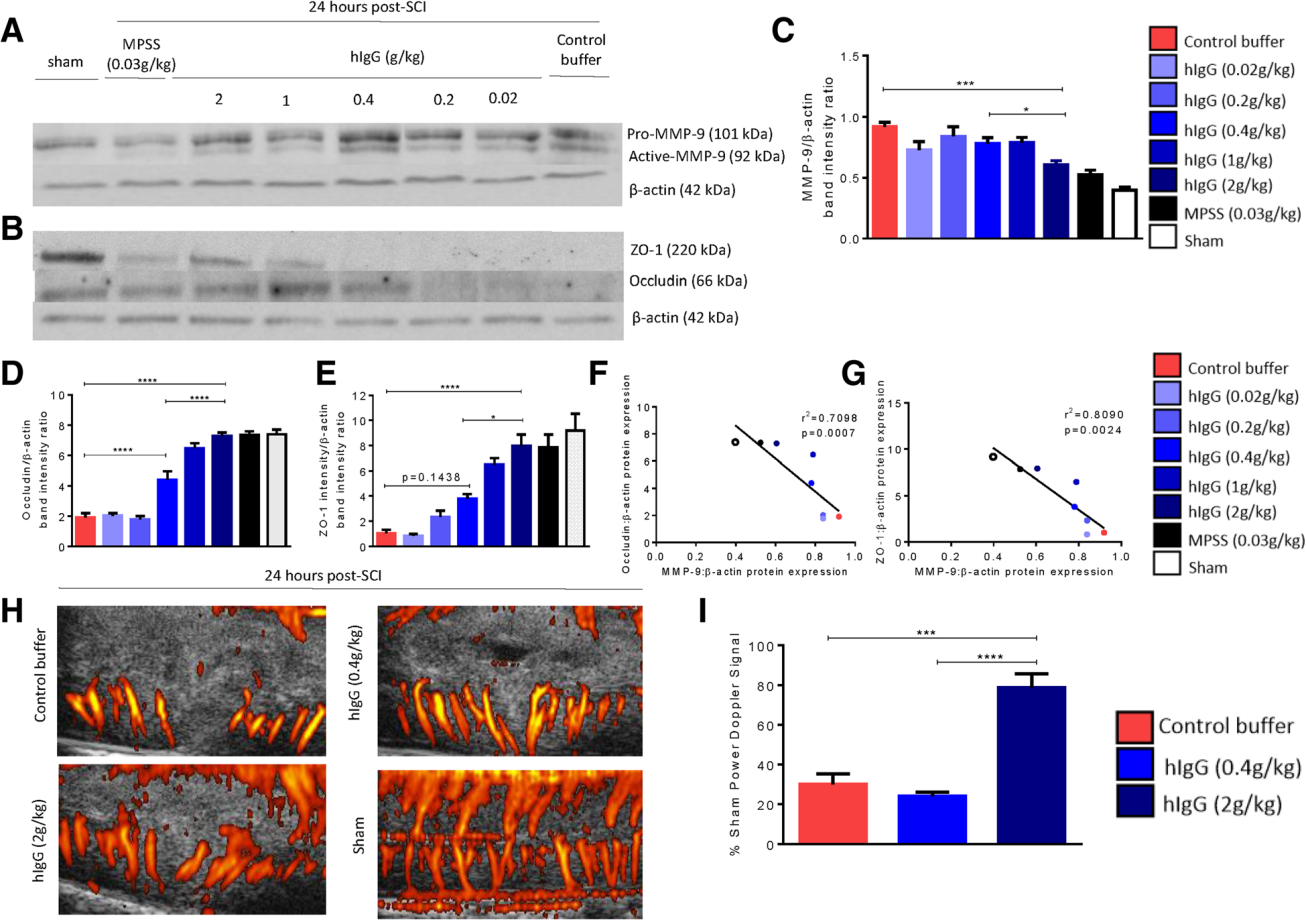
hIgG (2 g/kg) effects on the spinal cord neurovasculature 24 h after injury. **a** At 24 h post-SCI, active-MMP-9 and β-actin (loading control) levels in the rat spinal cord were determined by western blot. A representative image of the blot is shown. **b** Similarly, at 24 h post-SCI, ZO-1, occludin, and β-actin (loading control) levels in the rat spinal cord were determined by western blot. A representative image of the blot is shown. **c** Densitometric analysis of the signal ratio between active MMP-9 and β-actin indicated that administration of high-dose hIgG (2 g/kg) at 15 min post-SCI significantly decreased active MMP-9 expression (red arrow) relative to both control buffer and low-dose hIgG (0.4 g/kg). **d**, **e** Densitometric analysis of the signal ratio between occludin, ZO-1, and β-actin indicated that administration of high-dose hIgG (2 g/kg) at 15 min post-SCI significantly increased occludin and ZO-1 relative to both the control buffer and hIgG (0.4 g/kg). **f**, **g** There are significant negative correlations in protein expression of occludin and MMP-9 expression, as well as ZO-1 and MMP-9 expression. **h** Representative images of Power Doppler imaging for animals treated with control buffer, hIgG (0.4 g/kg), and hIgG (2 g/kg) are shown. **i** At 24 h post-SCI, functional blood flow was analyzed by Power Doppler imaging. The data presented are normalized to time-matched shams. hIgG (2 g/kg) significantly increased the functional blood flow relative to the control buffer and hIgG (0.4 g/kg). One-way ANOVA, Tukey’s post-hoc test (*p < 0.05, **p < 0.01, ***p < 0.001, ****p < 0.0001). Data are presented as mean ± SEM values

The western blots demonstrated that the presence of inflammatory conditions can cleave pro-MMP-9 (101 kDa) to become active MMP-9 (92 kDa) [25] (Fig. 3a). The literature indicates that peak expression of active MMP-9 at 24 h post-SCI coincides with maximum neutrophil infiltration [22, 26]. At 24 h post-SCI, there was a dose-dependent decrease in active MMP-9 expression, with a significant difference between hIgG (2 g/kg) and the control buffer (one-way ANOVA, *p* < 0.0001; Tukey’s post-hoc test, *p* = 0.0056) as well as between the control buffer and hIgG (0.4 g/kg) (one-way ANOVA, *p* < 0.0001; Tukey’s post-hoc test, *p* = 0.0241) (Fig. 3c). The greater expression of active MMP-9 corresponds to the degradation of tight junctions that disrupt the BSCB, which is shown by the decrease in the expression of tight junction proteins (occludin and ZO-1) (Fig. 3b). Dose-dependent increases in both occludin and zonula occludens-1 (ZO-1) were observed in western blot for both hIgG-treated (0.4 g/kg) and hIgG-treated (2 g/kg) samples relative to the control buffer (Fig. 3b). Quantification of occludin and ZO-1 protein expressions is found in Figs. 3 d and e (occludin: one-way ANOVA, *p* < 0.0001; Tukey’s post-hoc test, *p* = 0.0317 and *p* < 0.0001 respectively for hIgG (0.4 g/kg) and hIgG (2 g/kg); ZO-1: one-way ANOVA, *p* < 0.0001; Tukey’s post-hoc test, *p* = 0.0301 and *p* < 0.0001 respectively for hIgG (0.4 g/kg) and hIgG (2 g/kg)). Reduced MMP-9 activity is associated with a protective effect on ZO-1 and occludin, as there are significant negative correlations in the expression of active MMP-9/ZO-1 and active MMP-9/occludin (Figs. 3f, g). Loss and degradation of tight junction proteins have been implicated in the hyperpermeability seen in the BSCB after SCI.

This greater preservation of BSCB integrity prompted us to evaluate the effect of hIgG treatment on functional vascularity, which is a measure of active blood flow. Power Doppler imaging was performed to evaluate the functional blood flow at 24 h post-SCI (Fig. 3h, i). Relative to the control buffer, functional blood flow after SCI was improved with hIgG (2 g/kg) (one-way ANOVA, *p* < 0.0001; Tukey’s post-hoc test). In addition, functional blood flow was significantly improved between hIgG (0.4 g/kg) and hIgG (2 g/kg) (one-way ANOVA, *p* < 0.0001; Tukey’s post-hoc test, *p* < 0.0001). Representative images of functional blood flow are seen in Fig. 3h.

### hIgG significantly reduces neutrophil infiltration at 24 h post-SCI

**Fig. 4:**
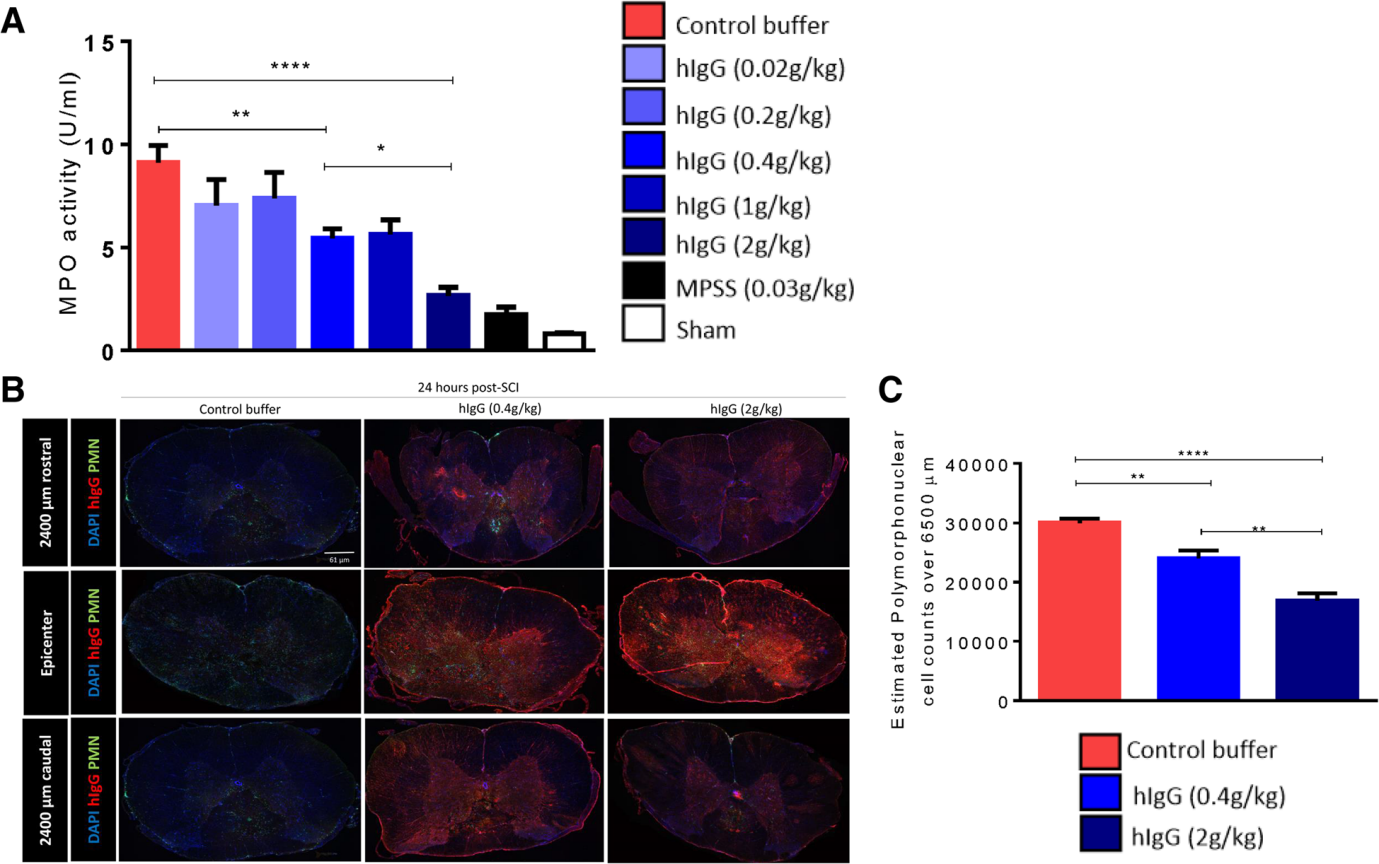
hIgG reduces neutrophil infiltration into the cord at 24 h post-SCI. **a** The MPO assay was used to detect MPO activity, which is an indirect measure of the neutrophil population in the spinal cord at 24 h post-SCI. Relative to the control buffer and hIgG (0.4 g/kg), high-dose hIgG (2 g/kg) administered at 15 min post-SCI significantly decreased MPO activity at 24 h post-SCI. **b** Representative fluorescence images demonstrate neutrophil infiltration and the presence of hIgG at the injury center and 2400 μm rostral and caudal in the spinal cord at 24 h post-SCI. Neutrophils are green (PMN) and overlapping with DAPI (blue), while hIgG is red. Scale bar represents 61 μm. **c** Relative to control buffer and hIgG (0.4 g/kg), stereological counts demonstrate significantly fewer neutrophils in animals treated with hIgG (2 g/kg). Furthermore, there was a significant difference between the animals treated with control buffer and hIgG (0.4 g/kg). One-way ANOVA, Tukey’s post-hoc test (*p < 0.05, **p < 0.01, ***p < 0.001, ***p < 0.0001). Data are presented as mean ±SEM values

The preservation of the spinal cord neurovasculature encouraged us to evaluate the changes in immune cell infiltration, as one physiological function of the BSCB is to limit the entrance of immune cells into the spinal cord [24]. Neutrophils are the first systemic immune cell to infiltrate after SCI, with the number of cells increasing between 3 and 6 h after injury, and peak infiltration observed at 24 h post-SCI [2, 22]. The literature has largely suggested that reducing neutrophil infiltration improves recovery in both rat and mouse SCI models [4, 5, 12, 15], although it has also been reported that neutrophil depletion worsens the neurological recovery after SCI [27]. Specifically, our laboratory and others have demonstrated that hIgG administration attenuates neutrophil infiltration into the cord after SCI [12, 15]. MPO is an enzyme found primarily in the azurophilic granules of neutrophils, and its activity is an excellent correlate to the absolute number of neutrophils in the cord. Thus, MPO is a specific and sensitive marker to quantify neutrophil infiltration into injured tissue [4, 15]. At 24 h post-SCI, MPO activity was found to be significantly higher in animals with SCI compared to sham animals. Our laboratory previously showed that hIgG (0.4 g/kg) significantly reduced MPO activity 24 h after SCI [15], and the present study reproduced this finding. Specifically, hIgG (0.4 g/kg) significantly reduced MPO activity as compared to the control buffer (one-way ANOVA, *p* < 0.0001; Tukey’s post-hoc test, *p* = 0.0019). Further, we showed that hIgG (2 g/kg) had superior immunomodulatory effects relative to both the control buffer and hIgG (0.4 g/kg) (one-way ANOVA, *p* < 0.0001; Tukey’s post-hoc test, *p* < 0.0001 and *p* = 0.0178, respectively). To complement the MPO assay, immunohistochemistry was performed to detect neutrophil infiltration at 24 h post-SCI, and unbiased stereology was used to quantify the infiltration in response to different hIgG doses (Fig. 4b). Importantly, in the injured rats treated with hIgG (2 g/kg), there were significantly fewer neutrophils found in the spinal cord relative to rats treated with hIgG (0.4 g/kg) and control buffer (one-way ANOVA, *p* < 0.0001; Tukey’s post-hoc test, *p* = 0.0022 and < 0.0001). Furthermore, relative to the control buffer, hIgG (0.4 g/kg) reduced neutrophil infiltration (one-way ANOVA, *p* < 0.0001; Tukey’s post-hoc test, *p* = 0.0072) (Fig. 4c). This is aligned with our previously published results [15].

### hIgG changes the expression of both inflammatory and anti-inflammatory cytokines in the spinal cord at 24 h post-SCI

**Fig. 5:**
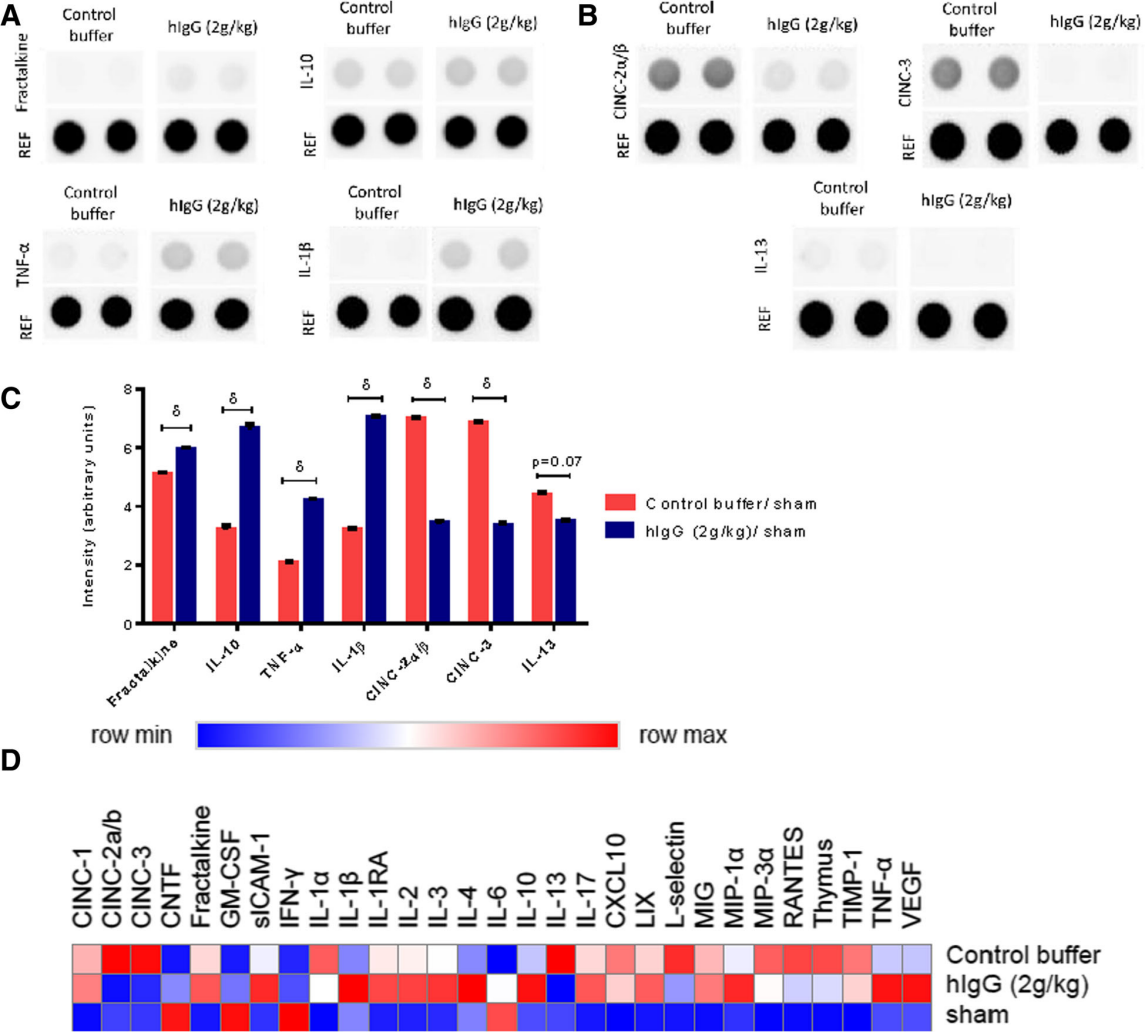
hIgG (2 g/kg) modulates inflammation in the spinal cord after SCI. **a**, **b** Representative images of the membrane for various cytokines in the injured spinal cord after treatment with either control buffer or hIgG (2 g/kg). Changes in cytokines were assessed with the R&D ELISA Proteome Profiler array, which compares the relative levels of 29 cytokines. **c** Changes to cytokines expressed in the injured spinal cord in response to treatment The protein levels were normalized to time-matched sham (laminectomy only) controls. Levels of IL-10, CX_3_CL1, IL-1β, and TNF-α were significantly higher after hIgG (2 g/kg) treatment, but levels of CINC-2α/β, CINC-3, and IL-13 were significantly lower. **d** Relative differences of the 29 cytokines analyzed by the R&D ELISA Proteome Profiler array are shown with a heat map generated using the BROAD Institute’s R implementation of Morpheus. Data are expressed as mean Log2 (fold change) and as indicated by scale bar, blue indicates minimum level (reduction) and red indicates maximum level (increase). Multiple *t* tests, Holm-Sidak correction was performed (^δ^*p* < 0.05). Data are presented as mean ± SEM values

To explain how hIgG (2 g/kg) can impose immunomodulatory effects on neutrophils, we sought to identify if these changes are associated with altered cytokine production in the injured spinal cord. A pro-inflammatory or anti-inflammatory microenvironment in the spinal cord can influence the recruitment, activation, and effector functions of immune cells. Using the R&D proteome profiler array (Fig. 5a), relative to control buffer, hIgG (2 g/kg) treatment altered the levels of both anti-inflammatory cytokines (interleukin (IL)-10, fractalkine (CX_3_CL1), IL-13) and inflammatory cytokines (IL-1β, TNF-α, CINC-2α/β, CINC-3) (multiple *t* tests; Holm-Sidak correction; IL-10, *p* = 0.0006; CX_3_CL1, *p* = 0.001; IL-1β, *p* = 0.002; IL-1β, *p* = 0.004; TNF-α, *p* = 0.0023; CINC-2α/β, *p* = 0.0032; CINC-3, *p* = 0.0029; IL-13, *p* = 0.07) (Fig. 5c). Representative images of the array membrane for respective cytokines are shown in Figs. 5a, b. The expression of the other cytokines detected by the R&D proteome profiler is demonstrated with a heat map (Fig. 5d).

### High-dose hIgG may interfere with immune cell trafficking by binding to vascular cell adhesion molecule-1

**Fig. 6:**
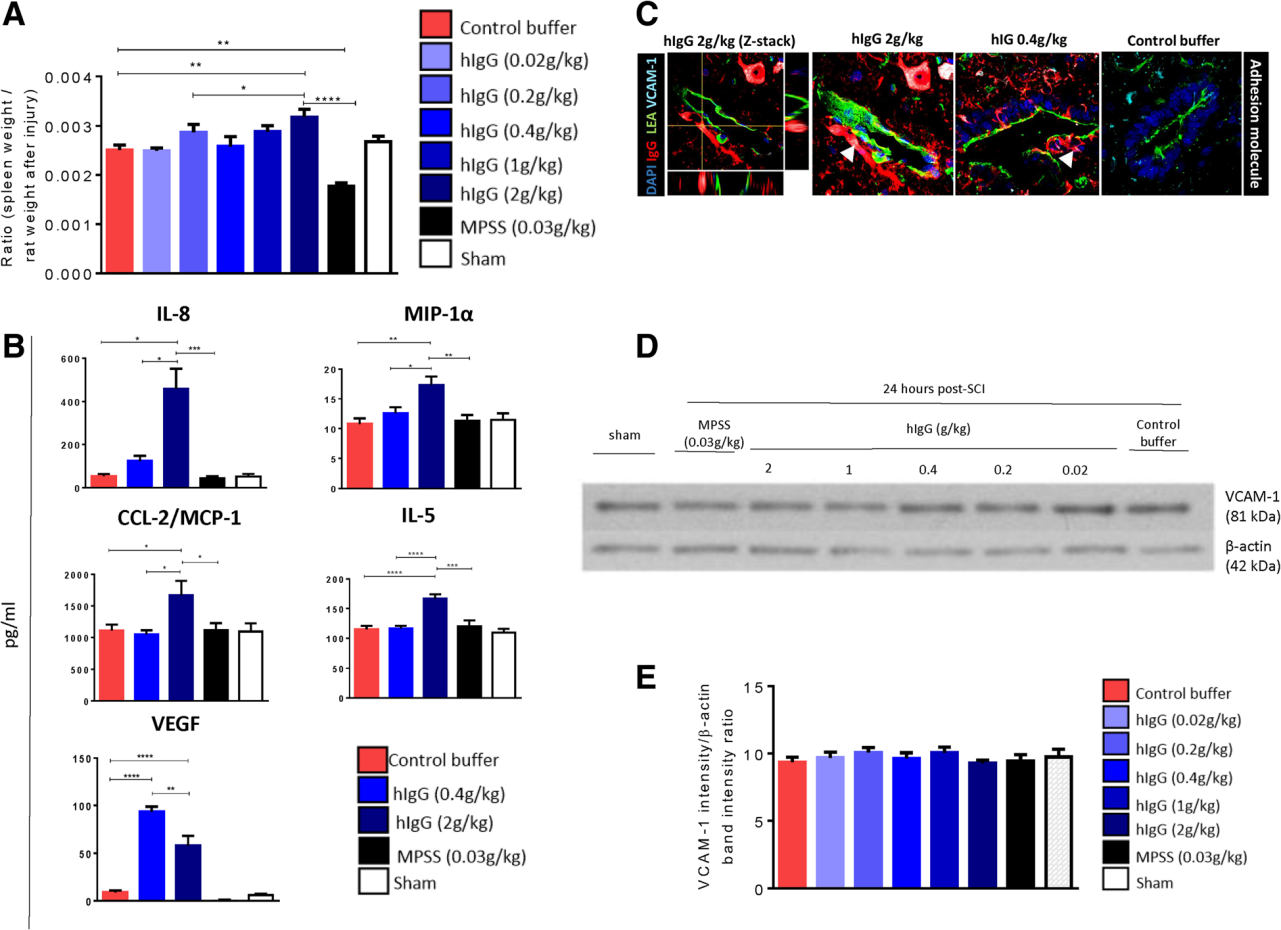
hIgG (2 g/kg) modulates serum cytokine expression and binds to vascular cell adhesion molecule-1. **a** At 24 h post-SCI, hIgG (2 g/kg) significantly increased the splenic weight, while MPSS (0.03 g/kg) decreased the weight. **b** hIgG (2 g/kg) enhanced the serum protein cytokine expression of numerous pro-inflammatory cytokines: IL-8, MIP-1α, CCL-2/MCP-1, and IL-5. VEGF was also upregulated. However, opposite to hIgG (2 g/kg), MPSS (0.03 g/kg) decreased the expression of these inflammatory cytokines and growth factor. **c** Representative confocal images (× 120) demonstrate that hIgG co-localizes with the spinal cord VCAM-1, with hIgG (red), blood vessels (LEA, green), and VCAM-1 (bright blue). Co-localization is indicated by white arrows. **d** A representative western blot of the spinal cord protein expression of VCAM-1 at 24 h post-SCI. **e** Densitometric analysis of the signal ratio between VCAM-1 and β-actin indicated that administration of high-dose hIgG (2 g/kg) at 15 min post-SCI failed to decrease VCAM-1 protein expression relative to the control buffer and hIgG (0.4 g/kg). One-way ANOVA, Tukey’s post-hoc test (*p < 0.05, **p < 0.01, ***p < 0.001, ****p < 0.0001). Data are presented as mean ± SEM values

At 24 h post-SCI, relative to control buffer and hIgG (0.4 g/kg), hIgG (2 g/kg) significantly increased the splenic weight (Fig. 6a). Conversely, MPSS (0.03 g/kg) significantly decreased the splenic weight. The spleen is a major source of immune cells after SCI, and gross structural changes in the spleen are adaptions to the immune response at acute time points [28]. As MPSS (0.03 g/kg) and hIgG (2 g/kg) had different effects on splenic weight, but both demonstrated anti-inflammatory effects at the injured spinal cord, this suggested potential changes in immune cell trafficking. Hence, protein expression of cytokines in serum after different treatments was evaluated. Using a commercially available rat cytokine array, we observed that hIgG (2 g/kg) upregulated IL-8, MIP-1α, CCL-2/MCP-1, and IL-5, while MPSS (0.03 g/kg) significantly downregulated the expression (Fig. 6b). Relative to the control buffer, vascular endothelial growth factor (VEGF) was also upregulated by hIgG treatment. However, of the hIgG-treated groups, expression was significantly lower in the hIgG (2 g/kg) group.

Significant changes in the serum cytokine expression prompted the evaluation of relevant adhesion ligands, which are a major component of immune cell trafficking into injured tissue. Ligands used by neutrophils, such as vascular cell adhesion molecule-1 (VCAM-1) [29], are notable as neutrophils are the main population of infiltrating immune cells at 24 h post-SCI [22]. With immunohistochemistry, we observed that hIgG co-localized with spinal cord VCAM-1 (Fig. 6c). However, despite the previously observed anti-inflammatory effects of hIgG, VCAM-1 protein expression in the spinal cord was not reduced by hIgG (2 g/kg) (Fig. 6d, e).

### High-dose hIgG significantly reduces lesion tissue volume at 24 h post-SCI, which translates to long-term neurobehavioral recovery and tissue preservation at 6 weeks post-SCI

**Fig. 7:**
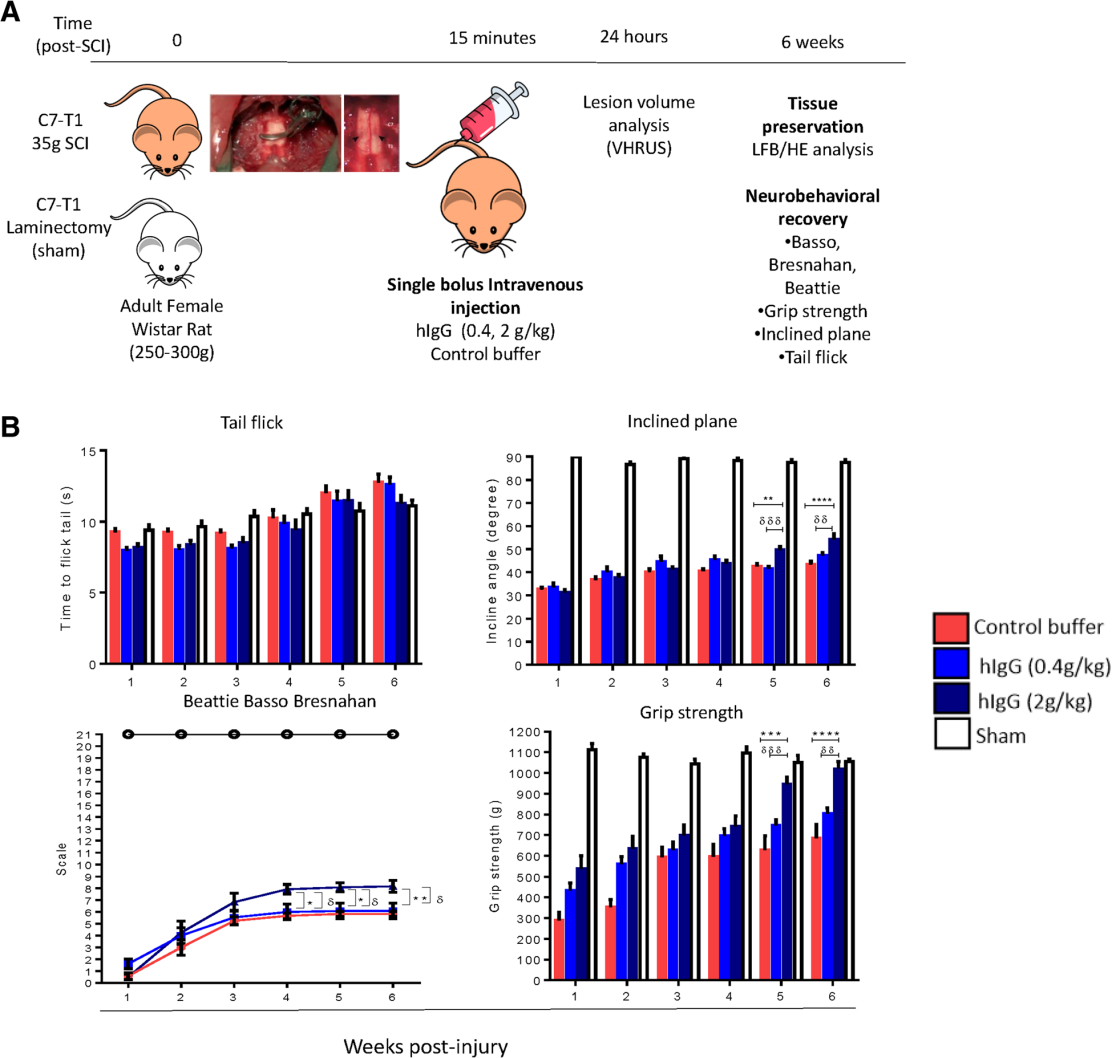
hIgG (2 g/kg) results in significant neurobehavioral improvement. **a** Illustration depicting the experiments and protocol applied. Adult female Wistar rats (250 to 300 g) received either a C7-T1 35 g clip-compression injury or sham surgery (C7-T1 laminectomy). At 15 min post-injury, a single bolus of hIgG (0.4 or 2 g/kg) or control buffer was administered to the injured rats through the tail vein. Functional recovery was assessed weekly by individuals blinded to the treatment groups for 6 weeks. **b** Relative to the control buffer and hIgG (0.4 g/kg), hIgG (2 g/kg) led to a significantly better improvement in the whole-body and trunk motor function, gross sensorimotor movement/hindlimb function, and grip strength as evaluated by the inclined plane, Beattie-Basso-Bresnahan, and grip strength, respectively. Improvements were mainly seen in weeks 4 and 5 post-SCI. hIgG had no effect on neuropathic pain after an injury. Two-way ANOVA, Tukey’s post-hoc test (*p < 0.05; **p < 0.01, ***p < 0.001, ****p < 0.0001 for comparisons between the control buffer and hIgG (2 g/kg), ^δ^*p* < 0.05, ^δδ^*p* < 0.01, ^δδδ^*p* < 0.001, ^δδδδ^*p* < 0.0001). Data are presented as mean ± SEM values

While hIgG (2 g/kg) had acute benefits by modulating the acute neuroinflammatory response at 24 h post-SCI, it was unknown if these molecular changes would translate into improved functional and tangible tissue preservation at both short-term (24 h) and long-term (6 weeks) time points post-SCI. In order to analyze the effects of hIgG on neurobehavioral functional recovery, rats with SCI receiving either control buffer, hIgG (0.4 g/kg), or hIgG (2 g/kg) performed weekly behavioral assessments up to 6 weeks post-SCI (Fig. 7a). Functional recovery was tested using the inclined plane, BBB Locomotor Scale, grip strength, and tail flick. hIgG (2 g/kg) animals performed significantly better than those treated with control buffer and hIgG (0.4 g/kg) (two-way ANOVA, *p* < 0.0001) (Fig. 7b). Improvements were mostly observed at weeks 4, 5, and 6 for these behavioral assessments. For the inclined plane, hIgG (2 g/kg) animals were observed to have improved whole-body and trunk motor function. The BBB indicated that hIgG (2 g/kg) animals had a greater hindlimb motion, suggesting that hIgG-treated (2 g/kg) animals had substantial weight support and extensive movement in all three joints, while control buffer and hIgG-treated (0.4 g/kg) animals had no weight support and less extensive joint movement. hIgG-treated (2 g/kg) animals also displayed greater forelimb grip strength. However, hIgG (2 g/kg) had no effect on improving the neuropathic pain, as indicated by a lack of differences observed in the tail flick test. Sham animals did not display functional deficits.

**Fig. 8:**
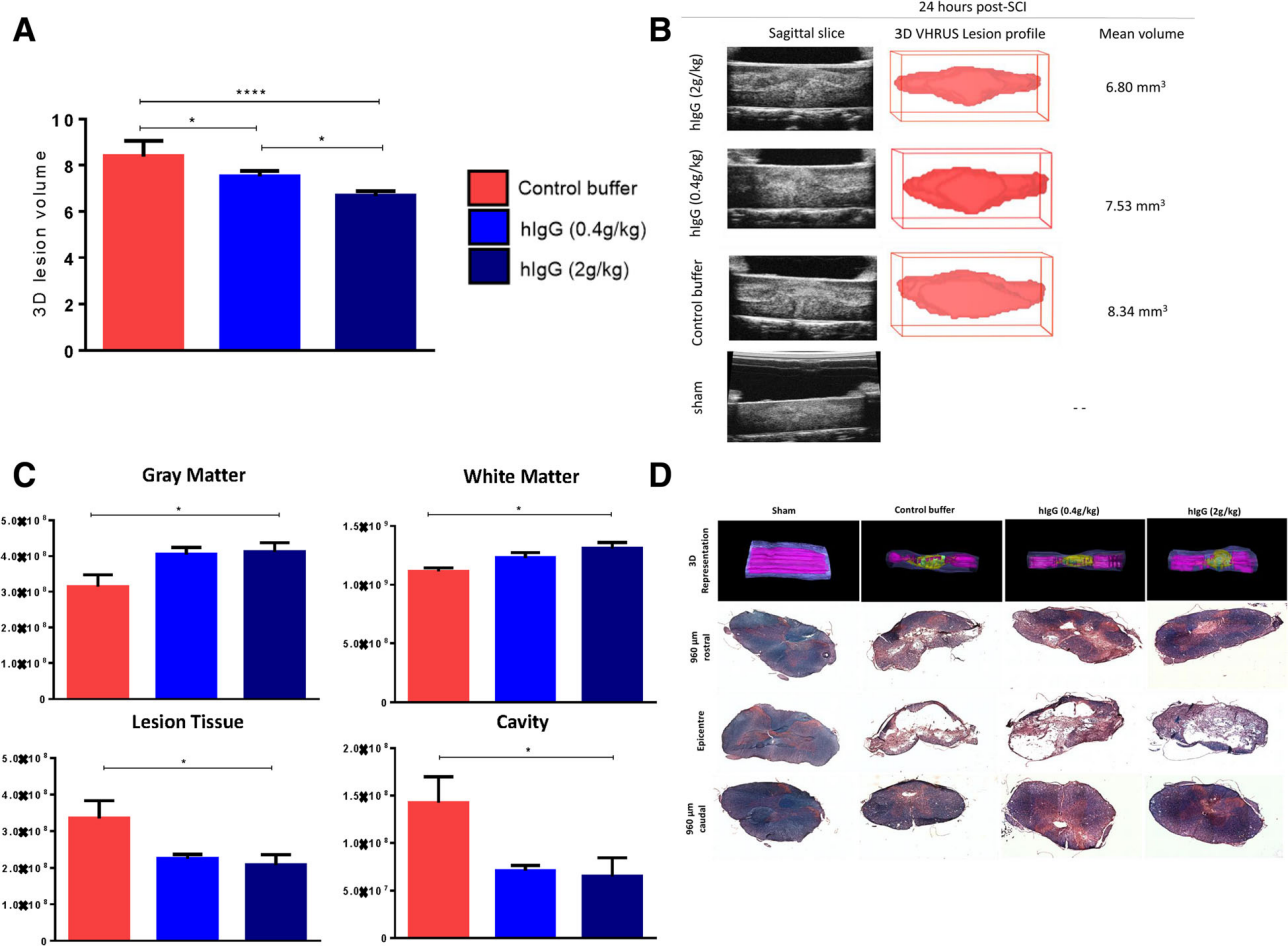
hIgG (2 g/kg) results in significant tissue preservation at both acute and chronic time points post-SCI. **a** At 24 h post-SCI, relative to the control buffer and hIgG (0.4 g/kg), hIgG (2 g/kg) significantly decreased the lesion volume in the injured spinal cord. **b** From top to bottom, representative images of the SCI lesion for animals treated with control buffer, hIgG (0.4 g/kg), hIgG (2 g/kg), and quantified volumes of the lesion are shown. In vivo very high-resolution ultrasound (VHRUS) imaging in B-Mode (11 × 7mm) was used to quantify the acute lesion volume. **c** At 6 weeks post-SCI, hIgG (2 g/kg) conferred protection by increasing the white and gray matter volumes, while concurrently decreasing the cavity and lesion tissue volumes. Significant improvements were only observed between the control buffer and hIgG (2 g/kg). **d** Representative 3D reconstructions of the spinal cords from sham and injured rats treated with control buffer, hIgG (0.4 g/kg), and hIgG (2 g/kg). Dark blue indicates white matter, purple indicates gray matter, while lesion and cavity are indicated by yellow and light blue, respectively. Images of LFB/HE-stained spinal cord tissue from all treatment groups and sham, with tissue sections from the epicenter, 960 μm caudal and rostral from the injury site. For VHRUS, one-way ANOVA Tukey’s post-hoc test (*p < 0.05, **:*p* < 0.01, ***p < 0.001, ****p < 0.0001). For LFB/HE, one-way ANOVA, Tukey’s post-hoc test (*p < 0.05, **p < 0.01, ***p < 0.001, ****p < 0.0001 for comparisons between the control buffer and hIgG (2 g/kg), ^δ^*p* < 0.05, ^δδ^*p* < 0.01, ^δδδ^*p* < 0.001, ^δδδδ^*p* < 0.0001). Data are presented as mean ± SEM values

In order to determine if acute immunomodulatory changes can lead to improved tissue preservation at acute and chronic time points, thus supporting neurobehavioral improvement, spinal cord histological analyses were performed at 1 day and 6 weeks post-SCI with in vivo VHRUS and LFB/HE staining, respectively. Using VHRUS, relative to both the control buffer and hIgG (0.4 g/kg), hIgG (2 g/kg) treatment was able to reduce the lesion volume (one-way ANOVA, *p* < 0.001; Tukey’s post-hoc, *p* < 0.0001 and *p* < 0.05, respectively) (Fig. 8a, b). There was also a reduction in lesion volume between the control buffer and hIgG (0.4 g/kg). A reduction in lesion volume can translate to increased tissue preservation. At 6 weeks post-SCI, LFB/HE quantification demonstrated hIgG (2 g/kg) was able to enhance tissue preservation by increasing the volumes of white and gray matters, as well as concurrently decreasing the lesion tissue and cavity volumes (Fig. 8c) (one-way ANOVA, *p* < 0.0001). While protection conferred by hIgG (2 g/kg) occurs both rostral and caudal of the epicenter, the protection was most evident at the caudal regions of the injured spinal cord. The preservation of white matter and gray matter, along with a concurrent reduction of the lesion and cavity, is demonstrated by representative LFB/HE images in Fig. 8d. Of note, the acute differences observed with VHRUS between the control buffer and hIgG (0.4 g/kg) were not detected with LFB/HE at 6 weeks post-SCI. Nevertheless, the preservation of tissue with hIgG is important, as it aligns with previous findings made by our laboratory and others [12, 13, 15].

## Discussion

In this study, a well-characterized model of SCI was used to carry out a dose-dependent examination of intravenously administered hIgG’s potential to attenuate neuroinflammation after SCI in rats. Our current results demonstrate that hIgG (2 g/kg) is more effective than hIgG (0.4 g/kg) to treat SCI and provides evidence that the immunomodulatory effects of hIgG are mediated through preserving the spinal cord neurovascular unit. Specifically, high-dose hIgG (2 g/kg) has significant protective effects on the spinal cord vasculature. These benefits are associated with reduced neutrophil infiltration, decreased expression of pro-inflammatory enzymes, and a dominant anti-inflammatory environment in the spinal cord. These effects translate into reduced lesion volume in the spinal cord and greater functional blood flow after SCI.

### High-dose hIgG for SCI treatment

In the healthy CNS, an intact BSCB limits the entry of large molecules (such as human immunoglobulin G) [15, 30]. As such, the normal concentration of human immunoglobulin G in a steady-state cerebrospinal fluid/serum ratio is 0.0027, with approximately 0.009 to 0.0017% of systemically administered immunoglobulin G reaching the healthy CNS. In the presence of CNS injury (i.e., SCI), a compromised BSCB allows human immunoglobulin G to enter into the CNS at a significantly increased. Here, we show that hIgG (2 g/kg) has significantly greater immunomodulatory effects than hIgG (0.4 g/kg) to treat SCI. Importantly, high-dose hIgG (2 g/kg) is used in other pre-clinical autoimmune disease models in both the peripheral and central systems, such as epilepsy [31], airway inflammation [32], chronic inflammatory demyelinating polyneuropathy [33], and idiopathic thrombocytopenic purpura [34].

### High-dose hIgG has effects on the spinal cord neurovascular unit

hIgG was found to co-localize with various components of the rat BSCB, such as astrocytes and pericytes, while accumulating in the vicinity of other components (blood vessels) and immune cells (microglia, neutrophils) of the spinal cord. Accumulation of hIgG due to the disrupted BSCB aligns with our previously published results [15]. The co-localization of hIgG on rat astrocytes and pericytes is of particular interest, as this suggests the ability of hIgG to modulate immune cell infiltration through the neurovascular unit [21]. Pericytes are an essential component of the BSCB and a sensor for neuroinflammatory signals produced by endothelial and parenchymal cells after CNS trauma [35, 36]. Of the 3 components in the spinal cord neurovasculature unit (astrocytes, pericytes, and vessels), the integrity of the BSCB is primarily determined by the extent of MMP-9 expression in pericytes after the inflammatory response. MMP-9 expression causes pericytes to detach and migrate away from the basal lamina and, hence, results in BSCB disruption. The role of pericytes in maintaining the barrier integrity under healthy conditions is seen when pericyte-deficient mice demonstrate barrier breakdown and hypoperfusion [37]. Interestingly, we show that hIgG (2 g/kg) significantly decreases MMP-9 expression. While the excessive proteolytic activity of MMPs is responsible for SCI pathophysiology, it should also be noted that MMP-9 activity is physiologically necessary for remodeling the extracellular matrix, tissue morphogenesis, and wound healing [24, 25].

An additional indicator of the protective effects of high-dose hIgG on the neurovascular unit is the preservation of tight junction proteins, as indicated by higher protein levels of occludin and ZO-1 after high-dose hIgG treatment. A more durable BSCB is beneficial, as reducing vascular damage prevents immune cells from permeating into the injured spinal cord, which effectively limits the spread of secondary injury cascades [38]. In our model of clip compression-contusion SCI, we have previously demonstrated that the BSCB is most disrupted at 24 h post-SCI and partially recovered by 2 weeks post-SCI. Importantly, this timeline corresponds with the period where there is a maximal MMP-9 activity, an enzyme whose biological substrates include multiple components of BSCB [24]. In the current paper, we provide evidence that hIgG (2 g/kg) significantly reduces vascular permeability at a period of time post-SCI where the BSCB is most compromised. The greater integrity of the BSCB and improved functional vascularity are potential downstream effects of the interaction between hIgG and the spinal cord neurovascular unit, while also being associated with reduced active MMP-9 expression. As there is a positive correlation between BSCB integrity, functional vascularity, and behavioral/tissue recovery after SCI [38, 39], the neuroprotective interactions of hIgG-astrocytes-pericytes merit further investigation. Early repair of the BSCB has important implications for the treatment of SCI.

### High-dose hIgG has significant modulatory effects on immune cell populations after SCI

Neutrophils increase the extent of damage when entering into the injured tissue by binding to various adhesion molecules expressed on the inflamed endothelium, such as VCAM-1 [40]. Previous research has employed various methods to decrease neutrophil infiltration after SCI, demonstrating both beneficial and harmful effects. Thus far, pre-clinical studies for SCI have used antibodies to block neutrophil entry [4, 5, 27, 41–43]. This highlights that strict immunosuppression can impede recovery, as it indiscriminately eliminates both beneficial and detrimental aspects of neuroinflammation. Our finding that hIgG significantly reduces neutrophil infiltration (using MPO assay and PMN stereology) is supported by previous publications [12, 15]. Reduced neutrophil infiltration, in combination with the observed improvements in BSCB integrity, suggests a feedforward protective loop. While a more intact BSCB can limit neutrophil infiltration, those neutrophils that successfully migrate into the inflamed tissue can continue to perform pathological effector functions [24]. Reduced neutrophil infiltration after hIgG (2 g/kg) confers further protection by decreasing MMP-9 expression, effectively limiting leukocyte entry through the compromised BSCB. It is noteworthy that the ability of hIgG to reduce the expression of MMP-9 has been previously reported [44], but the mechanism is ill-defined. Furthermore, it was observed that hIgG co-localizes with VCAM-1, potentially suggesting that hIgG may impede the interactions between VCAM-1 and very late antigen-4 (VLA-4) [29], which would further limit neutrophil infiltration. Previous reports have demonstrated that blocking VLA-4 with monoclonal antibodies significantly reduces neutrophil infiltration in a mouse model of stroke and improves outcomes [45]. Furthermore, with in vitro inflammatory conditions, high-dose hIgG mediates immunomodulatory effects through interfering with leukocyte adhesion and rolling mechanisms [46, 47].

### High-dose hIgG modulates the inflammatory environment after spinal cord injury

Here, we report a change in the expression of cytokines in the injured spinal cord after hIgG (2 g/kg) treatment. Cytokines are the main orchestrators of the neuroinflammatory response, both of which are small families of proteins capable of recruiting and activating the aforementioned immune cells after SCI [48].

The ability of hIgG to promote an anti-inflammatory environment after SCI has been previously reported by our laboratory, as hIgG (0.4 g/kg) has been shown to reduce inflammatory cytokines IL-1β, IL-6, and monocyte chemoattractant protein-1 at 4 h post-SCI [15]. In the current study, we demonstrate that, relative to the control buffer, hIgG (2 g/kg) increases protein expression of both inflammatory and anti-inflammatory cytokines. This phenomenon may be attributed to the administration of a high dose of an immunomodulatory molecule derived from an organism different from the host, which can elicit a modest immune response due to multiple mechanisms, such as the activation of complement system, receptor clustering and activation on innate immune effector cells, and recruitment of immune cells. Past studies have indicated that polymorphisms and interspecies differences of hIgG influence the binding of IgG to Fcγ receptors (FcγRs) and may alter the downstream signaling cascades [49, 50], a limitation of the current study that warrants further research. Nevertheless, the immune response elicited when administering an exogenous molecule can lead to greater expression of IL-6 and TNF-α [51], a change observed in the current study. In vitro models indicate that hIgG can inhibit migration of neutrophils across endothelial cells stimulated with TNF-α and IL-1β [52], another phenomenon similar to what is reported in this manuscript. Reduction of neutrophil infiltration is also associated with decreased levels of neutrophil chemoattractants CINC-2α/β and CINC-3 after hIgG (2 g/kg) treatment [53].

Of note, the increased IL-1β after hIgG (2 g/kg) treatment can bind to the immune cell receptors and activate the p38 pathway to enhance transcription and translation of multiple cytokines [54]. These include TNF-α and CX_3_CL1, both of which are highly expressed after hIgG (2 g/kg) treatment. Importantly, the anti-inflammatory effects of hIgG (2 g/kg), through increased IL-10 and CX_3_CL1, can offset the inflammatory effects of IL-1β and TNF-α [55]. Furthermore, IL-10 favors a beneficial anti-inflammatory environment after neurotrauma by reducing MPO activity [56], edema, tissue damage, and apoptosis [57–59]. In regard to CX_3_CL1, it may exist in two forms and have different functions on immune cells expressing the corresponding receptor, CX_3_CR1 [60]. CX_3_CL1 is expressed mainly by neurons but can also be expressed by astrocytes under inflammatory conditions. In its membrane-bound form, CX_3_CL1 is an adhesion molecule for CX_3_CR1^+^ cells. However, under excitotoxic conditions (such as SCI), CX_3_CL1 can be cleaved into its soluble form, bind to CX_3_CR1^+^ cells, and maintain them in a quiescent state after injury [60–62]. As IL-10 can increase the levels of CX_3_CR1 mRNA [63], the greater levels of IL-10 may also mediate anti-inflammatory effects through a similar mechanism. Our findings of anti-inflammatory effects, whether they are directly or indirectly caused by high-dose hIgG, align well with the past literature. These include many mechanisms, such as reduction of integrin activation, modulation of the expression and function of FcγRs, neutralization of autoantibodies, regulation of cell proliferation, interference with activation of the complement cascade, and modulation of the cytokine network [10, 64].

In stark contrast, the administration of hIgG (2 g/kg) significantly upregulated multiple pro-inflammatory cytokines in sera after SCI, a phenomenon that has been reported in patients with immune disorders [65–67]. Here, we report that hIgG (2 g/kg) increased serum protein expression of neutrophil chemoattractants (IL-8, MIP-1α, CCL-2/MCP-1) and eosinophil activator (IL-5). Notably, pro-inflammatory cytokines are expectedly downregulated after MPSS (0.03 g/kg) treatment [68]. As SCI patients experience systemic immune deficiency [7], the ability of hIgG to enhance pro-inflammatory mediators may dampen the severity of immune suppression by mobilizing innate and adaptive immune cell populations that are reduced in pre-clinical models of SCI and SCI patients [7, 69]. Changes in cytokine secretion and immune cell effector functions correspond to alterations mediated by F_c_ and F_(ab)2_ components of hIgG [70, 71]. Specifically, the increase in inflammatory cytokines may traffic immune cells to the spleen, a major immune cell reservoir/source that's role in SCI pathophysiology has gained increased attention recently [72]. Neutrophils are found in the rat spleen and active after SCI [73, 74]. As hIgG (2 g/kg) alleviates neutrophil infiltration in the spinal cord and increases splenic weight, the increased levels of neutrophil chemoattractants may redirect neutrophils to the spleen. Future studies are needed to evaluate how hIgG modulates splenic immune cell populations after SCI.

### High-dose hIgG significantly increases tissue preservation and functional recovery after SCI

Tissue preservation and neural repair after SCI are important, as the spinal cord is a complex structure with a large number of ascending and descending neural tracts that connect specific brain structures to precise locations in the body. In this study, we extend our previous findings, as well as others [12, 13, 15], by showing that hIgG (2 g/kg) results in significantly more potent immunomodulatory effects as mediated by acute molecular and biochemical changes. This translates into better functional recovery and tissue preservation at both acute and chronic time points post-SCI. As enhancing the survival of 5–10% of the fibers at the lesion center of the spinal cord helps drive the segmental circuits involved in the production of basic locomotion [75], relatively small improvements in neuroanatomical integrity after SCI can have a substantial, clinically relevant impact on neurological recovery and greatly reduce patient morbidity.

## Conclusion

Extensive pre-clinical and clinical evidence has shown that hIgG therapy is safe, and adverse effects associated with its administration are mild and transient. Although MPSS is the only pharmaceutical neuroprotective therapy currently used for SCI in the clinic, it is potentially suboptimal due to the systemic immune deficiency exhibited by SCI patients and the dual nature of the acute inflammatory response. In the current paper, we show that high-dose hIgG is an easily deliverable therapeutic at the acute stage of SCI, and the neuroprotective effect is mediated, in part, by preserving BSCB integrity. This attenuates immune cell infiltration, promotes an anti-inflammatory environment, and enhances tissue preservation and functional recovery after injury. However, before hIgG can be used in the clinic to treat SCI, further optimization of efficacy and delineating its mechanism are required. This may include studying more clinically relevant time points to administer hIgG as well as determining which component of the hIgG molecule mediates the observed immunomodulatory effects. Overall, our study supports the growing trend of using immunomodulatory, rather than immunosuppressive, strategies to treat SCI.

## Data Availability

The datasets used and/or analyzed during the current study are available from the corresponding author on reasonable request.

## Abbreviations

ANOVA: Analysis of variance
BBB: Beattie-Basso-Bresnahan
BSCB: Blood-spinal cord barrier
CX_3_CL1: Fractalkine
CX_3_CR1: Fractalkine receptor
FcγR: Fcγ receptor
hIgG: Human immunoglobulin G
HRP: Horseradish peroxidase
Iba-1: Ionized calcium-binding adapter molecule-1
IL: Interleukin
LFB/HE: Luxol fast blue/hematoxylin and eosin
MMP-9: Matrix metalloproteinase-9
MPO: Myeloperoxidase
MPSS: Methylprednisolone
mRNA: Messenger ribonucleic acid
PBS: Phosphate-buffered saline
SCI: Spinal cord injury
SEM: Standard error of mean
T-BST: Tris-buffered saline with 0.2% Tween
TJ: Tight junction
VCAM-1: Vascular cellular adhesion molecule-1
VEGF: Vascular endothelial growth factor
VHRUS: Very high-resolution ultrasound
ZO-1: Zonula occludens-1
